# Alcoholic Liver Disease among Patients Admitted to the Department of Internal Medicine of a Tertiary Care Centre: A Descriptive Cross-sectional Study

**DOI:** 10.31729/jnma.7434

**Published:** 2022-04-30

**Authors:** Ashlesha Chaudhary, Arun Kumar Chaudhary, Aashutosh Chaudhary, Abashesh Bhandari, Sujata Dahal, Suzit Bhusal

**Affiliations:** 1 Nepal Medical College and Teaching Hospital, Jorpati, Kathmandu, Nepal; 2 Department of Statistics, Nepal Commerce Campus, Tribhuwan University, Minbhawan, Kathmandu, Nepal; 3 Kathmandu University School of Medical Sciences, Dhulikhel, Kavre, Nepal; 4 Department of Internal Medicine, Nepal Medical College and Teaching Hospital, Jorpati, Kathmandu, Nepal; 5 Patan Academy of Health Sciences, Lagankhel, Lalitpur, Nepal; 6 Kathmandu Medical College and Teaching Hospital, Sinamangal, Kathmandu, Nepal

**Keywords:** *alcoholic liver diseases*, *jaundice*, *liver function test*, *Nepal*

## Abstract

**Introduction:**

Alcoholic liver diseases comprise a spectrum of liver diseases including fatty liver, alcoholic hepatitis, and cirrhosis. Diagnosis at advanced stages is frequent for this condition and data regarding the prevalence of such patients at early stages are limited. The aim of this study is to find out the prevalence of alcoholic liver disease among patients admitted to the Department of Internal Medicine of a tertiary care centre.

**Methods:**

A descriptive cross-sectional study was conducted among 538 patients admitted to the Department of Internal Medicine of a tertiary care centre in Nepal between 3^rd^ November, 2021 and 22^nd^ February, 2022 after receiving ethical approval from the Institutional Review Committee of the hospital (Reference number: 006-078/079). Convenience sampling was done. Data were collected and entered in Microsoft Excel and analysed using Statistical Package for the Social Sciences version 24.0. Point estimate at 95% Confidence Interval was calculated along with frequency and percentage for binary data along with mean and standard deviation for continuous data.

**Results:**

Among 538 patients, alcoholic liver disease was seen in 42 (7.80%) (5.53-10.07 at 95% Confidence Interval). The mean age of the patients was 53.85±10.88 years. Among these patients 25 (59.52%) were males and 17 (40.47%) of them were females.

**Conclusions:**

Our study showed that the prevalence of alcoholic liver disease was lower as compared to similarly reported literature.

## INTRODUCTION

Alcoholic Liver Disease (ALD) comprises a broad spectrum of liver diseases encompassing a wide range from asymptomatic or early stages to Alcoholic Steatohepatitis (ASH) and advanced ALD. The early stages include fatty liver or alcoholic steatosis whereas, advanced ALD is characterized by alcoholic hepatitis, cirrhosis and its complications.^1^ Alcohol is a well-established hepatotoxin^2^ but proper studies regarding alcohol and liver diseases are still lacking among the Nepalese population. The aim of this study is to find out the prevalence of

alcoholic liver disease among patients admitted to the Department of Internal Medicine of a tertiary care centre.

## METHODS

A descriptive cross-sectional study was conducted from 3^rd^ November, 2021 to 22^nd^ February, 2022 in the Department of Internal Medicine of Nepal Medical College and Teaching Hospital, Attarkhel, Jorpati, Kathmandu, Nepal. The study was started after obtaining ethical approval from the Institutional Review Committee of the hospital (Reference number: 006-078/079). All patients admitted to the internal medicine ward of the hospital with an age of more than 20 years were included in the study. Informed consent was signed to indicate the patient's understanding and permission to include them in the study. Convenience sampling was done.

The sample size was calculated by using the formula:

n = (Z^2^ × p × q) / e^2^

  = (1.96^2^ × 0.50 × 0.50) / 0.05^2^

  = 385

Where,

n = minimum required sample size
Z = 1.96 at 95% of Confidence Interval (CI)
p = prevalence of alcoholic liver disease, 50%^3^
q = 1-p
e = margin of error, 5%

After adding 10% to address the non-response rate, a total sample size of 427 was obtained. However, 538 patients were enrolled in this study.

The patients who consented to the study were enrolled in the study and history was taken from them during the performance of clinical examination. The patients were subjected to Complete Blood Count (CBC), Ultrasonography (USG), Liver Function Tests (LFT), and ascitic fluid analysis. The diagnosis of alcoholic liver disease was made based on hepatic steatosis on ultrasound and/or elevation in liver enzymes i.e. Aspartate Aminotransferase (AST), Alanine Aminotransferase (ALT), serum bilirubin <3 mg/dl, and the absence of other causes of liver disease.^1^ Among the patients with signs and symptoms suggestive of upper gastrointestinal bleeding, upper gastrointestinal endoscopy was done to rule out oesophagal varices.

The diagnosis of Spontaneous Bacterial Peritonitis (SBP) was established based on positive ascitic fluid bacterial cultures and the detection of an elevated absolute fluid Polymorphonuclear Neutrophil (PMN) count in the ascites (>250/mm^3^) without an evident intra-abdominal surgically treatable source of infection.^4^ Hepatic encephalopathy was diagnosed after proper history and physical examination to identify the cognitive deficits and neuromuscular impairments characteristic of hepatic encephalopathy after exclusion of other causes of mental status changes and evaluation of possible precipitating factors.^5^

Data regarding demography, clinical features, laboratory findings, and complications were collected and entered in Microsoft Excel and analysed using Statistical Package for the Social Sciences version 24.0. Point estimate at 95% Confidence Interval was calculated along with frequency and percentage for binary data along with mean and standard deviation for continuous data.

## RESULTS

**Figure 1:**
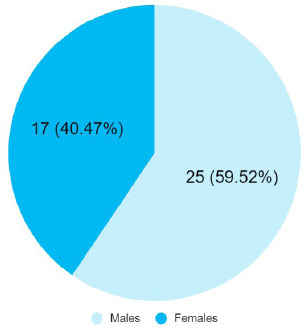
Gender-wise distribution of the patients with alcoholic liver disease (n= 42).

Among 538 patients, alcoholic liver disease was seen in 42 (7.80%) (5.53-10.07 at 95% Confidence Interval). The mean age was 53.85±10.88 years and the condition was more prevalent in males 25 (59.52%) (Figure 1).

The total serum bilirubin levels were increased in the majority of the patients with mean serum bilirubin of 4.90±5.37. The AST/ALT ratio was more than 1 in 37 (88.09%) and more than 2 in 15 (35.71%) patients with alcoholic liver disease with a mean of 2.28±1.69. The Serum Albumin Ascites Gradient (SAAG) was >1.1 g/dl in all of the patients with ascites with a mean value of 2.12±0.62 (Table 1).

**Table 1:** Laboratory parameters of the patients with alcoholic liver disease (n= 42).

Laboratory Tests	(Mean±SD)
Haemoglobin	10.79±3.10
Total serum bilirubin	4.90±5.37
Aspartate Transaminase (AST)	105.33±68.16
Alanine Aminotransferase (ALT)	62.92±69.66
AST/ALT ratio	2.28±1.69
Alkaline Phosphatase (ALP)	159.02±78.88
Serum albumin	3.06±0.73
Ascitic albumin	0.51±0.18
Serum Albumin Ascites Gradient (SAAG)	2.12±0.62
Ascitic protein	1.15±1.41
Prothrombin Time (PT)	18.76±6.41
International Normalised Ratio (INR)	1.79±2.02

With regards to the clinical features of the patients, the most common features were icterus and ascites. Whereas, spontaneous bacterial peritonitis was seen in only 2 (4.76%) (Table 2).

**Table 2:** Clinical features of patients with alcoholic liver disease (n= 42).

Features	n (%)
Icterus	28 (66.67)
Ascites	28 (66.67)
Spontaneous Bacterial Peritonitis (SBP)	2 (4.76)
Coagulopathy	6(14.28)
Esophageal varices	25 (59.52)
Hepatic encephalopathy	8(19.04)

## DISCUSSION

Alcoholic Liver Disease (ALD) is one of the main causes of chronic liver disease worldwide and the patients usually present to the hospital after they have developed jaundice or have symptoms suggestive of the complications of cirrhosis.^6^ There is very little knowledge regarding the prevalence of this condition in our population and the patients usually present late with complications.

Based on our study, the prevalence of alcoholic liver disease among patients presenting to our tertiary care centre was 7.80% among 538 patients admitted to the internal medicine department of the hospital which was low when compared to similar studies done in other tertiary care centres of Nepal.^3,7,8^ The mean age of the patients from our study was comparable to these studies. With regards to the gender-wise distribution of the patients, our study showed that ALD was more predominant in males which is similar to other studies.^3,9^ Similarly, our study reported that the mean serum values of serum bilirubin levels, AST and ALT were increased in the majority of the cases which held true in other studies as well which could indicate hepatocellular damage.^3,10^

AST/ALT ratio has been widely studied over the past few decades into using this ratio for the diagnosis of alcoholic liver disease separate from other forms of liver diseases. The common fact about these enzymes is that they require pyridoxal-5'-phosphate (Vitamin B6) for proper functioning. Heavy alcohol consumers are found to be nutritionally deficient, affecting the production of ALT more than AST leading to a rise in the AST/ALT ratio.^11,12^ The ratio should normally be <1. In our study, the AST/ALT ratio was more than 1 in majority (88.09%) and more than 2 in 15 (35.711%) patients with a mean of 2.28±1.69. However, in patients with alcoholic liver disease, the ratio has been found to be >1 in around 92% of the patients and >2 in around 70% of the patients. Hence, the increase in the ratio >2 is found to be strongly suggestive of alcoholic liver disease.^11^

The Serum Ascites Albumin Gradient (SAAG) is calculated by the difference between the albumin level of serum and of ascitic fluid which may be used to assess the extent of ascites and indicate the presence of portal hypertension if SAAG is ≥1.1 g/dl, but reports have been in contrast.^13-15^ In our study, the SAAG was ≥1.1 g/dl in all of the patients with ascites with a mean value of 2.12±0.62. The SAAG is thought to reflect the colloid osmotic pressure gradient and the degree of portal hypertension.^13^

The results of the study cannot be generalised as the population under study is limited to patients admitted to the tertiary care centre only. Also, because of the descriptive nature of this study, an association between exposure and outcome cannot be made in this study design and risk factors cannot be made out.

## CONCLUSIONS

Our study showed that the prevalence of alcoholic liver disease in tertiary care centres was lower as compared to similarly reported literature. This condition has been increasing in females. And, the patients usually present at a late stage which warrants the need for early diagnosis and management of this condition. Most of the biochemical parameters at an initial stage could be of paramount importance in guiding the treatment.
